# The Absence of Educational Grades

**Published:** 1921-04-23

**Authors:** 


### NURSES WANT A CAREER.*

### II.—The Absence of Educational Grades

The nurse, even if she is an optimist, can scarcely hope for a career until some radical changes take place. The most obvious of these is educational. It is a remarkable fact that every profession except nursing demands of its entrants a certain minimum educational attainment. *Vide* the Regulations for the Bar, the Solicitors' profession, Medicine, Pharmacy, Dentistry, and Veterinary Surgeons. Moreover, the tendency is to raise the standard in every case in accord with modern progress. Education is compulsory: it may be availed of free or at nominal cost. Members of a profession have a certain status to maintain, and hence the necessity to keep out those whose attainments are so lacking as to bring their calling into contempt. Into the nursing profession all and sundry may enter. The matron of the training-school is the judge of fitness, and she, poor woman, has very often no choice.

It may seem to be a fetish of mine, but I am bound to observe here that in their reckless pursuit after registration a great deal of mischievous propaganda was disseminated by its advocates. The "one portal" cry had the unavoidable effect of minimising the importance of education. It was described as democratic, and as such obtained a certain cheap popularity, but there is no doubt that it tended to degrade, especially in view of the extraordinary fact that at this portal no questions are asked as to general education. I am disposed to the opinion that there is more behind this fact than appears on the surface. I heard a very distinguished medical man who is actively concerned with the training of nurses confess that he had no desire to see educated women entering; that there was always the tendency for them to think they "knew too much." This indicated a terrible bias, one which I am afraid cannot be dissociated from jealousy. I mention it as an explanation, in part at all events, of the absence of opposition to the one-portal doctrine, a doctrine which played directly into the hands of those who endeavour to keep nursing in a sunless region.

The consequence of the absence of educational essentials is obvious in many ways. In the first place, the attitude of the Ministry of Health to nurses in providing the nation with health visitors is so marked as to be inexplicable on any other than educational grounds. The one body of people whose training and occupation naturally fit them for health visiting are for all practical purposes shut out. Then the providing for the welfare of industrial workers is a rapidly extending movement, and is being taken up by the Universities. But the nurse is not mentioned. She stands outside the gate, and may remain at her post without hope of betterment. There can be no room for doubt that the lack of an educational standard for entrants constitutes a formidable barrier to progress.

Then those who would, if they could, remedy this defect find themselves confronted with another difficulty. Even with an open door where there are no questions asked there is a scarcity of applicants, and one may inquire if it is reasonable to turn away those who offer themselves as candidates because their English composition and arithmetic are deficient. Here we have a sort of vicious circle. If uneducated candidates are unwilling to come forward, how can it be hoped to attract those who are able to pass an examination?

I am afraid that until the truth is thoroughly ventilated, not only amongst the public but even amongst nurses, no substantial change is likely to be made. Only last week I read an editorial in the *Irish Times* congratulating the promoters of Registration on the fact that the hitherto ill-requited profession of nursing will now become a "close" profession, and that salaries will automatically rise. This was written in perfectly good faith, and was a natural inference from an article supplied to the Editor, also published. But, being entirely untrue, it is most pernicious, and calculated to lead the public to believe that the compiling of a list of names will affect the wage market.

There are many potent influences working together to the detriment of the nurse. But, so far as I am able to judge, the most serious is the educational defect. This operates prejudicially, subjectively, and objectively. It lessens her value in her own estimation and in the estimation of the public. It debars her from being able to compete with others who lack technical training and skill, and, worst of all, it makes entrance to the nursing profession possible to people who are not far removed from illiteracy.

If nurses had a career, in the proper sense of the term, to look forward to one condition would be the existence of educational grades, with an established minimum. Diplomas could be issued, including extra subjects which would make the holders possible candidates for health visitorships, welfare inspectors, and so forth, and thus there would always be an incentive to endeavour. One naturally associates the College of Nursing with academic distinction. It seems to me that the possibilities of the educational field are wide and the necessities very pressing. But unless there is a strong body of public nursing opinion in its favour the outlook is not very promising. IERNE.

* The previous article appeared on April 9.

